# The human gut pan-microbiome presents a compositional core formed by discrete phylogenetic units

**DOI:** 10.1038/s41598-018-32221-8

**Published:** 2018-09-19

**Authors:** Daniel Aguirre de Cárcer

**Affiliations:** Departamento de Biología, Universidad Autónoma de Madrid, Madrid, Spain

## Abstract

The complex community of microbes living in the human gut plays an important role in host wellbeing. However, defining a ‘healthy’ gut microbiome in terms of composition has remained an elusive task, despite its anticipated medical and scientific importance. In this regard, a central question has been if there is a ‘core’ microbiome consisting of bacterial groups common to all healthy humans. Recent studies have been able to define a compositional core in human gut microbiome datasets in terms of taxonomic assignments. However, the description of the core microbiome in terms of taxonomic assignments may not be adequate when considering subsequent analyses and applications. Through the implementation of a dynamic clustering approach in the meta-analyisis of comprehensive 16S rRNA marker gene datasets, this study found that the human gut pan-microbiome presents a preeminent compositional core comprised of discrete units of varying phylogenetic depth present in all individuals studied. Since both microbial traits and ecological coherence show signs of phylogenetic conservation, this outcome provides a new conceptual framework in the study of the ecosystem, as well as important practical considerations which should be taken into account in future research.

## Introduction

The gut microbiome, the community of microbes living in the large intestine, provides a variety of services relevant to host well-being. Defining what constitutes a normal gut microbiome in healthy individuals is regarded as pivotal^1^ for the development of predictive models for diagnosis and management of maladies linked to microbiome dysbiosis. Unfortunately, the strong variability in gut microbiome composition persistently observed across individuals has hindered efforts towards this goal. Hence, whether there is a ‘core’ microbiome, consisting of bacterial groups common to all healthy humans^2^, remains a central issue in the field^3^.

There have been previous studies describing concrete efforts to identify a compositional core of the human intestinal microbiome. The initial studies^4,5^ were not able to detect species-level core groups shared among all individuals. More recent efforts were able to detect a core in terms of taxonomic assignments^1,6^. However, taxonomic assignments are heavily biased towards well sampled groups^7^ and describe groups of varying phylogenetic depth and unknown boundaries. Hence, the description of the core microbiome in terms of taxonomic assignments may not be adequate when considering subsequent analyses and applications.

Sekelja *et al*.^8^ carried out an innovative meta-analysis of previous studies that was not based on predefined taxonomic or similarity thresholds. Instead, they used a principal components analysis (PCA) to describe the similarities between sequences, and defined as core microbiome the set of minimal portions of the reduced similarity space containing sequences from all individuals. In this manner, they observed two prevalent core groups related to the Lachnospiraceae family. However, the analysis was based solely on the first two axes of the PCA, representing merely 24.2% of the total variation in the original space. Therefore, their approach could potentially have grouped sequences that are actually more dissimilar when considering the complete similarity space. Moreover, such approach necessarily involves the loss of core groups that are not well modeled by the first two components of the PCA, as acknowledged by the authors.

The present study analyzes the human gut pan-microbiome in terms of 16S rRNA OTUs present in all individuals, where such OTUs have been produced dynamically over a range of similarity clustering thresholds, as opposed to using a single arbitrary fixed threshold or pooled taxonomic rank assignments, which so far had represented the dominant praxis in the field. The approach employed herein is analogous to that employed by Sekelja *et al*., in that it produces a set of minimal portions of the similarity space populated by sequences present in all individuals. However, the propose approach is not biased nor limited by the use reduced-space ordination. The approach was applied in the meta-analysis of three large datasets^9–11^ to uncover, among other things, that in all three cases the human gut pan-microbiome can be understood as having a compositional core comprised of several discrete portions of the phylogenetic space.

## Methods

### Datasets

Three sets of 16S rRNA gene sequences (obtained using primer pair F515-R806 targeting the V4 hypervariable region) derived from human stool samples were employed (Table 1); Global Gut dataset^10^ (Rural Malawi, Metropolitan U.S., and Venezuela Amerindians. Illumina GAIIx, 100 bp sequences), TwinsUK dataset^9^ (mostly female U.K. residents, including 171 monozygotic and 245 dizygotic twin pairs, as well as 183 subjects with BMI > 30. Illumina MiSeq, 250 bp sequences), and LifeLines dataset^11^ (Dutch population-based cohort. Illumina MiSeq, 250 bp sequences). For each dataset the same individual sample selection criteria was applied: only one sample per subject, subjects were healthy according to each study’s procedures, subjects were >3 years old, and with no antibiotic treatment in the 6 months period prior to sampling. Finally, for each dataset a few samples meeting all criteria were removed due to a relative low sequencing depth.

**Table 1:** Datasets’ characteristics.

Name	Geographic distribution	Number of individuals	Sequence depth	Read length	Sequencing technology
Global	Malawi, USA, Venezuela	382	>300.988	100 bp	Illumina GAIIx
TwinsUK	UK	977	>14.975	250 bp	Illumina MiSeq
LifeLines	Netherlands	884	>14.398	250 bp	Illumina MiSeq

The datasets analysed during the current study are available from their original source^9–11^. Additional result files and scripts are available from the corresponding author on reasonable request.

### Sequence processing

The same procedures were carried out for all three datasets. Unless otherwise noted, QIIME (v1.9.1)^12^ scripts were employed during sequence processing. First, each dataset was subsampled to its maximum common depth to reduce computing effort. Then, chimeric sequences were identified (*usearch61*) and later removed. This procedure yielded >14.975, >300.988, and >14.398 sequences per sample for TwinsUK, Global, and LifeLines datasets, respectively. Subsequently, sequences were clustered into OTUs (*de novo* clustering using *usearch61*^13^) at all 0.01 steps between 0.97 and 0.75. At each step (except the initial 0.97 step) the representative sequences obtained from the previous step were employed as input (Multi-step OTU picking, http://qiime.org/tutorials/chaining_otu_pickers.html). Then OTU tables were produced for all clustering thresholds.

### Core OTU detection

**Figure 1:**
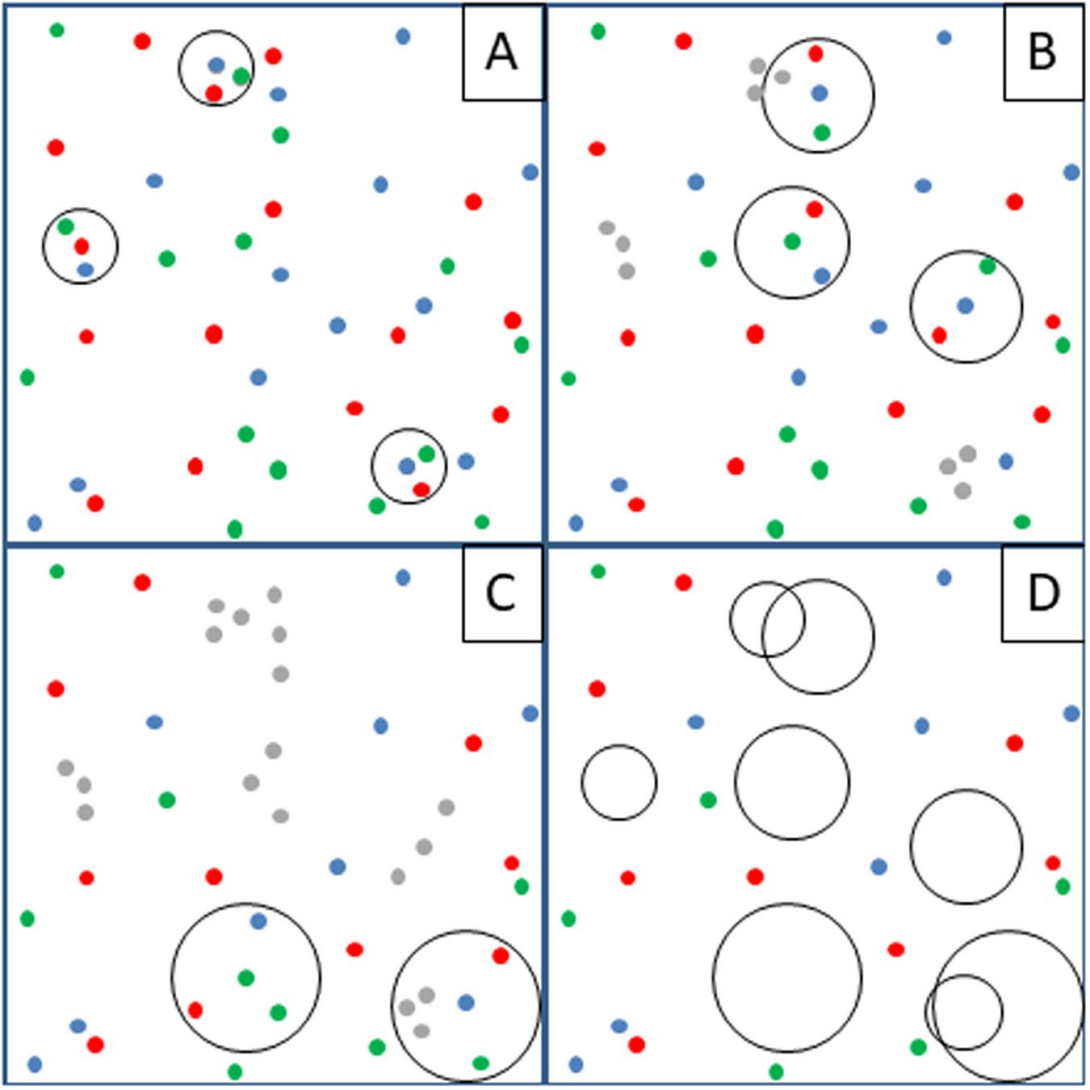
Graphical representation of the dynamic phylogenetic core identification approach. Panels in the cartoon provide a bidimensional representation of an imaginary N-dimensional sequence space populated by sequences (dots) from three different subjects (red, blue, green). During the initial iteration **(A)**, sequences are clustered into OTUs at the highest similarity threshold, and OTUs containing sequences from all subjects are designated as “core OTUs” (here depicted as circles). In the second iteration **(B)**, all sequences belonging to previously detected core OTUs are removed (grey dots), the remaining sequences are clustered into OTUs using a lower similarity threshold, and new OTUs containing sequences from all subjects are again designated as “core OTUs” (circles, now with larger radius). Subsequent iterations **(C)** continue in the same fashion. The result of the procedure **(D)** is a set of discrete portions of the sequence space (i.e. the core OTUs, depicted as circles) detected in all individuals and with different estimated phylogenetic depth (i.e. different radius sizes), jointly referred as the phylogenetic core.

In order to assess the existence of a phylogenetic core within each dataset the following procedure was undertaken (Fig. 1); for each clustering threshold (all 0.01 steps between 0.97 and 0.75), OTUs present in 100% of the samples were designated as ‘core OTUs’ only after removing all sequence data belonging to core OTUs detected at higher similarity clustering thresholds. The present study evaluated the existence of core OTUs along a 0.97–0.75 similarity range. The highest value was chosen as it is often employed as a “species-level” proxy. On the other hand, the lowest value was chosen as it is generally considered that nucleotide alignments degrade below this point.

### Statistics

Basic statistics for the OTU tables and core OTUs were obtained using various R functions (R core team, 2016) from packages *biom* and *matrixStats*. Empirical cumulative distributions were computed for each clustering threshold in order to compare each core OTU’s abundance and coefficient of variation values against those of all OTUs detected at the same threshold.

In order to explore the statistical significance associated with the detection of core OTUs at each clustering threshold, the following permutation test was carried out; first 100 randomized matrices were produced by shuffling all intra-subject values using the randomizeMatrix function from package *picante*^14^, hence maintaining sample richness (number of OTUs per sample). Then, for each randomized matrix the number of OTUs present in 100% of the subject was recorded, providing a sampling distribution. The ranking of the actual number of core OTUs at each threshold among its respective shuffled distribution provides the p-value. Hence, the approach measures the probability of obtaining that many core OTUs for each clustering threshold, given the particular structure of the community table.

### Taxonomic description of results

For each core OTU, all sequences belonging to such OTU were clustered into new OTUs at 0.97 similarity threshold (*de novo* clustering using *usearch61*) and a representative sequence from each novel OTU was kept. Then, *mothur* (v1.39.0) scripts^15^ were used for the taxonomic assignment of each representative sequence using SILVA_v123 reference database^16^ and the default 80% cutoff, and later to produce consensus taxonomic assignments for each core OTU (classify.otu). Finally, resources from R packages *ape*^17^ and *metacoder*^18^ were used for the graphical representation of results as metadiversity plots.

### Core OTUs vs. deposited bacterial genomes

First, all bacterial genomes data from ncbi’s RefSeq collection were downloaded (>60.000 genomes, 28-9-2016). Then, the longest 16S rRNA gene sequence existing for each genome was obtained. Later, sequences not spanning the region being compared were removed. Subsequently, *bowtie2* short read aligner^19^ was employed to map each of the representative sequences of OTUs defined at 0.97 distance for each core OTU against the 16S sequences derived from the bacterial genomes. Subsequent parsing of the alignment files provided, for each core OTU, the similarity between each representative sequence and the closest 16S sequence from a deposited genome, and such values were then translated into average and standard deviation values for each core OTU.

### Predicted metagenomes

Processed sequences belonging to the Global dataset (complete), and to each of the Global dataset core OTUs were employed. For each group of sequences, sequence names were modified to contain a single sample identifier, hence the Global dataset and each of the individual core OTUs datasets would be treated as individual metagenomes. Then, all sequence sets were individually clustered into OTUs using Greengenes^20^ 0.97 representative sequences (May 2013) as reference with *usearch61*. Subsequently, PICRUSt scripts^21^ were employed to transform OTU abundances into KO abundances (KEGG Orthology^22,23^), and later KO abundances were categorized into the different levels of the KEGG pathway hierarchy. The values obtained were transformed into relative abundances, and differences between each core OTU predicted metagenome and the complete Global predicted metagenome were expressed as fold change (FC) by dividing their respective relative abundance value for each hierarchical category. Only instances within the “Metabolism” category and showing a FC > 3 were considered.

## Results

Three sets of 16S rRNA gene sequences derived from human stool samples were analyzed (Table 1); The “Global” dataset^10^ (>320.000 sequences per subject; 382 subjects from rural Malawi, metropolitan U.S., and Venezuela Amerindians), “TwinsUK” dataset^9^ (>20.000 sequences per subject; 977 individuals, U.K.), and “LifeLines” dataset^11^ (17.000 sequences per subject; 884 individuals, The Netherlands). All three datasets were produced by different research groups and with differing protocols. Thus, in the present study all analyses were carried out independently for each dataset since microbial composition estimates may not be comparable among them^24^. In order to assess the existence of a phylogenetic core (Fig. 1), sequences were clustered into OTUs at all 0.01 steps between 0.97 and 0.75 similarity thresholds. Then, for each clustering threshold, OTUs present in 100% of the subjects were designated as ‘core OTUs’ only after removing all sequence data belonging to core OTUs detected at higher similarity clustering thresholds. In this manner, the approach produces a set of discrete minimal portions of the similarity space populated by sequences present in all subjects (i.e. the core OTUs), which serves as an adequate proxy for the phylogenetic core of the pan-microbiome.

Each of the datasets showed evidence of a phylogenetic core characterized by a varying number of core OTUs detected over a wide range of sequence similarity depths (Table 2). Computer simulations (i.e. intra-subject shuffling of OTUs abundance values) assessing the possibility of at least obtaining one core OTU by chance indicate that such possibility was for all datasets and clustering thresholds low (p < 0.01), which stems from the large number of observations (OTUs) and sparsity of the community tables (Suppl. Table 1). Sequencing depth and number of subjects likely influenced the phylogenetic core detected. In this regard, reducing the sequencing depth of Global dataset to that of LifeLines produced only one core OTU at 0.97 similarity, instead of 17. On the other hand, reducing LifeLines dataset to the same number of subjects as that of Global detected the shallowest core OTU at 0.93 similarity, instead of 0.89. This study focuses primarily on the results derived from the Global dataset, which, despite its smaller cohort size, is much more comprehensive in terms of distribution, lifestyle, and ethnicity (also featuring a much higher sequencing depth), meaning that it is more informative when assessing the human gut pan-microbiome compositional core.

**Table 2:** Summary of Phylogenetic Cores.

Dataset	Frequency	Range	Core groups per clustering threshold
TwinsUK	28.9 ± 11.6%	4.3–78.2%	94^1^, 92^1^, 91^2^, 90^2^, 89^1^, 88^1^, 87^3^, 85^2^, 80^1^
Global	65.6 ± 10.9%	33.2–91.2%	97^17^, 95^3^, 93^1^, 92^4^, 91^10^, 90^16^, 89^7^, 88^18^, 87^10^, 86^12^, 85^14^, 84^13^, 83^9^, 82^5^, 81^11^, 80^6^, 79^1^, 78^3^, 76^1^, 75^1^
LifeLines	22.4 ± 9.0%	2.0–66.7%	89^1^, 83^1^, 78^1^, 75^1^

Frequency; average pooled abundance of members of the core OTUs across the dataset. Range; minimum and maximum pooled abundance of the core OTUs across the dataset. Core groups per clustering threshold; Numbers represent similarity clustering thresholds (x10^−2^) were core OTUs were detected, and superscript values indicate the number of such OTUs observed for each threshold.

The Global core was characterized by 162 sequence clusters (i.e. core OTUs) present in all subjects, and detected along the entire clustering range employed (Table 2). Significantly, the Global core included 17 phylogenetically shallow OTUs at 0.97 similarity. Throughout the dataset, the core groups accounted for a large percentage of the total sequences (per-subject average 65.6 ± 10.9%; range 33.2–91.2%). The core OTUs showed very high relative abundances (all among the 10% most abundant OTUs of their respective clustering threshold) and high stability (i.e. low coefficient of variation) across the pan-microbiome (Suppl. Table 1). Interestingly, the most abundant core OTUs (average relative abundance >1%) are dominated by phylogenetically shallow (0.97 similarity) groups mostly taxonomically assigned to the Lachnospiraceae family, but also including putative members of the Ruminococcaceae and Bifidobacteriacea families. On the other hand, the remaining abundant core OTUs represent groups within the Bacteroidetes phylum which have been defined as core at mid-range similarity clustering thresholds (*ca*. 0.86).

**Figure 2:**
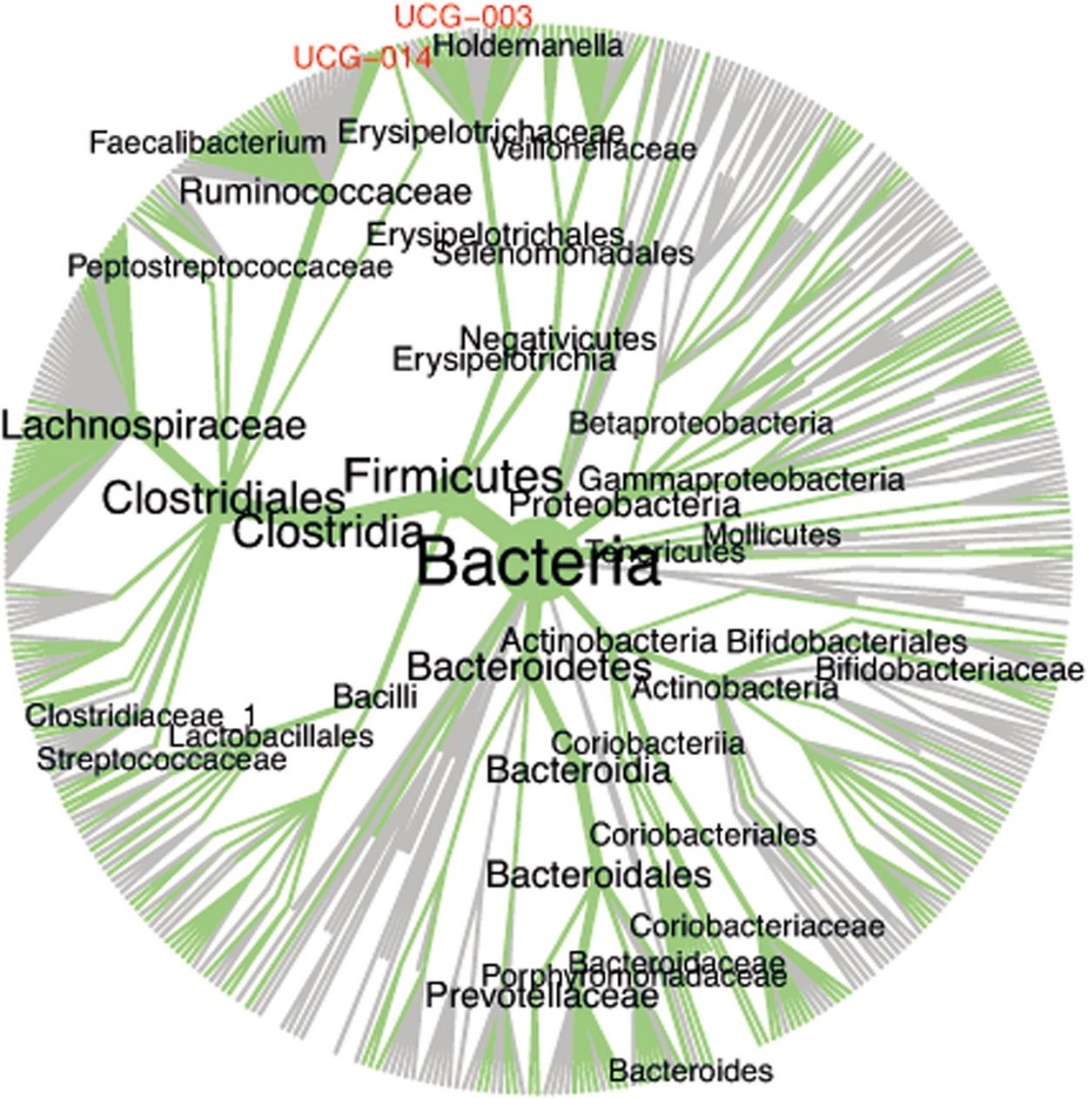
Taxonomic representation of the Global phylogenetic core. The Metadiversity Plot summarizes the taxonomic assignments obtained for all sequences in the Global dataset, were both label and node sizes correlate with each specific taxon’s abundance. Only the most abundant taxa are labeled. Taxonomic ranks not appearing as assigned to any within-core OTUs representative sequence (0.97 similarity) appear in gray and red. The figure provides an indication of the taxonomic breadth of the phylogenetic core.

Putative taxonomic assignment of members of the human gut phylogenetic core (Fig. 2) revealed that sequences within the core groups were most often affiliated to the Lachnospiraceae, and Ruminococcaceae-assigned groups were also abundant. Bacteroidetes-affiliated groups were also observed, mainly populated by Bacteroidacea-like sequences. Within this phylum, core OTUs populated by Prevotellaceae-assigned sequences were also detected, and sequences designated as Porphyromonadaceae and Rikenellaceae were also part of deep-lineage core OTUs. Additionally, some core OTUs were defined as Actinobacteria (Bifidobacteriaceae and Coriobacteriaceae), Lactobacillales, Erysipelotrichaceae, Veillonellaceae, and Proteobacteria (mostly Gamma-proteobacteria). However, putative taxonomic assignments at times produced conflicting results (not shown), especially below the 0.90 similarity threshold (approx.). Initially, these apparently conflicting results were observed as OTUs formed by sequences affiliated to different families within the same order. However, as the clustering threshold lowered, some OTUs were found with sequences assigned to different clades within the same phylum, and in some instances assigned to different phyla (Fig. S1). Such conflicting affiliations should represent true taxonomic breadth within an OTU, although they could also represent lack of congruency between taxonomic assignments and phylogeny, or even clustering pitfalls.

**Figure 3:**
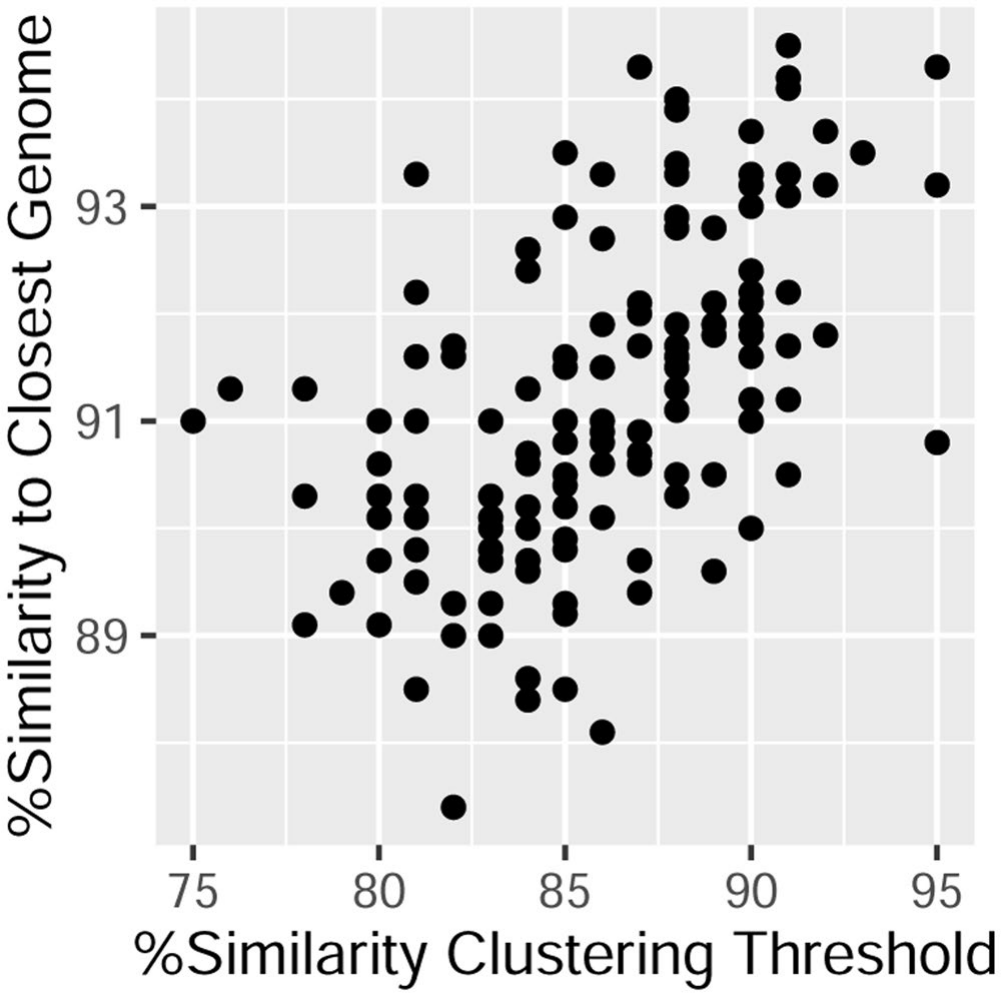
The phylogenetic core vs. sequenced genomes. The chart describes each core group detected in the Global dataset in terms of its phylogenetic depth (x axis) and its average within-core OTU similarity to closest sequenced genome (y axis).

To assess how well current reference genome collections capture the diversity contained within the human gut phylogenetic core, all within-core OTU representative sequences (defined at 0.97 similarity) were compared to 16S sequences derived from NCBI’s RefSeq genomes collection. The results (Suppl. Table 1) indicated that most of the 17 phylogenetically shallow core OTUs have closely related genomes sequenced (>0.97 similarity between both 16S sequences), the exception being two core OTUs affiliated to the Subdoligranulum (Ruminococcaceae) and Fusicatenibacter (Lachnospiraceae) genera which should become a target for genome sequencing projects. With regards to the deeper-lineage core OTUs (Fig. 3, Suppl. Table 1), the average within-core OTU similarity to a sequenced genome was surprisingly low (range 0.874 to 0.945), with an overall trend correlating average similarity and clustering threshold. The most extreme case was that of a core OTU defined at 0.95 similarity were average within OTU similarity to a sequenced genome was 90.8 ± 3.4. Luckily, a closely related genome was very recently sequenced^25^, hence providing some functional context for that group.

For the complete Global dataset and each of its detected core groups a prediction of functional composition of corresponding metagenomes was obtained using PICRUSt. PICRUSt represents a computational approach which has shown its usefulness in the prediction of the abundance of gene families in human gut microbiomes on the basis of 16S marker gene data^21^. The predicted metagenome for each core group was compared to that of the Global (complete) predicted metagenome, and results were limited to differences (FC >3) between metabolic pathways. A total of 442 differences were found (Additional file 1). Such differences were detected within all 11 sub-categories observed for the Global dataset, but concentrated in *Xenobiotics Biodegradation and Metabolism* (93), *Lipid Metabolism* (77), *Glycan Biosynthesis and Metabolism* (74), *Biosynthesis of Other Secondary Metabolites* (62), *Metabolism of Terpenoids and Polyketides* (53), and *Metabolism of Cofactors and Vitamins* (30). When categorized as particular pathways, these differences were observed in only 61 out of the 146 pathways (KEGG hierarchy level 3) detected for the Global predicted metagenome.

The most abundant core groups were attributed to the Firmicutes and Bacteroidetes phyla. Interestingly, the former included the highest similarity core phylogenetic groups detected, while the latter presented core OTUs defined at mid-range thresholds. This fact represents a very interesting result; while it could be related to different evolution rates between these phyla, or the fact that conservation depth may vary among traits, it may also imply some fundamental difference in the characteristics of the niches that these phyla occupy. This is especially so taking into account that trait conservation across the tree of life seems to occur in a hierarchical fashion, probably linked to biochemical complexity^26^. The former (10 core groups) showed 1.3 ± 0.99 differences with respect to the predicted metagenome for the Global dataset, while the latter (4 core groups) showed 10.2 ± 0.95 such differences. The few differences observed for these Firmicutes core groups were related to *Biosynthesis of Other Secondary Metabolites*, *Metabolism of Terpenoids and Polyketides*, *Lipid Metabolism*, and *Xenobiotics Biodegradation and Metabolism*. In the case of the mentioned Bacteroidetes core groups, differences were found for the same categories, with the exception of *Xenobiotics Biodegradation and Metabolism*, but also for *Metabolism of Cofactors and Vitamins*, and *Glycan Biosynthesis and Metabolism*.

## Discussion

This study describes a compositional core in the human gut pan-microbiome in terms of 16S rRNA sequence clusters obtained at different similarity thresholds. The rationale behind the approach employed is that each OTU serves as proxy to a phylogenetic group, where the similarity threshold provides a representation of the lineage’s depth. In this regard, while it is commonly accepted that OTUs may not convey exact phylogenetic coherence^27^, they are overwhelmingly employed as descriptors of microbial diversity, as their use allows us to overcome the biases associated with the use of bacterial taxonomic assignments as sequence grouping factor.

The strategy employed brings up the issue of how to appreciate the physiological and ecological diversity captured within the core OTUs when such OTUs are defined at varying similarity thresholds. In this respect, several studies have convincingly substantiated the idea that both microbial traits and ecological coherence are phylogenetically conserved, even in deep-linage clades^26,28–30^. Although it is beyond the scope of the present study, the proposed approach would benefit from further research aiming to establish which clustering method better preserves the phylogenetic relationships among sequences^31^, as well as the similarity limit below which OTUs loose biological or technical congruency. On the other hand, OTUs could be substituted by nodes in a phylogenetic tree^32^. Such approach should produce an enhanced phylogenetic resolution of the core groups, with the likely downside of escalating computing requirements and/or having to resort to the use of reference phylogenetic trees, with the (possible) concomitant loss of information.

In this work, the definition of a core OTU as “an OTU present in all individuals” is applied as an effective heuristic to discover potentially important phylogenetic groups within the Phylogenetic Core conceptual framework introduced herein. Clearly, the observed composition of a phylogenetic core will vary if the prevalence threshold varies, as illustrated by Huse *et al*.^4^ who explored how different prevalence thresholds affected the recovery of “core” phylotypes (0.97 similarity) from the Human Microbiome Project 16S dataset.

Here we have used the tried-and-tested *usearch61* clustering algorithm^13^. Do to the lack of true transitivity in the clustering of 16S sequences, the use of different algorithms may translate into the detection of phylogenetic cores with slightly differing compositions. On the other hand, differences in initial seeding between clustering runs could produce a similar effect. However, this should not jeopardize the validity of the Phylogenetic Core approach exemplified in this work, nor should it modify substantially the results.

The rules that govern microbial community assembly are often probed in terms of two opposing theories^33^: Neutral theory, where stochastic forces dominate the assembly, and Niche theory, where deterministic interactions between individuals, populations and the environment determine community composition. The gut microbiome represents a complex microbial community which is assembled *de novo* after birth. Hence, if stochastic forces were to dominate assembly, the core groups should most likely feature enhanced immigration-related characteristics, such as increased dispersal and(or) colonization abilities, high abundance within the metacommunity, or both. The increased immigration capabilities hypothesis is supported by the fact that members of the Ruminococcaceae and Lachnospiraceae families (predominant in the detected core) have recently been shown to produce resilient spores, specialized for host-to-host transmission^34^. On the other hand, the second hypothesis is reminiscent of global patterns in bacterial biogeography showing that cosmopolitanism correlates with high abundance in individual assemblages^35^, as well as the results presented here showing high overall abundance values for members of the core.

Despite these circumstantial facts, there is accumulating evidence supporting that community assembly in the gut is, to a certain extent, deterministic^36–39^. This fact, along with the abovementioned idea that microbial traits are phylogenetically conserved, brings us to the alluring hypothesis that the existence of phylogenetic core groups may relate to specific niches within the gut environment, and occupancy of such niches would require a specific set of phylogenetically conserved traits. On the other hand, the existence of members of the healthy gut microbiota which do not seem to belong to any core group may relate to neutral processes, but also to the occupancy of niches requiring sets of traits not showing strong phylogenetic signals. The results presented herein indicating that individual core groups are enriched in particular predicted metabolic functions, and jointly detected in only a subset of the metabolic functionalities detected for the complete pan-micribiome seem to align with the hypothesis. The use of this hypothesis as a conceptual framework in the study of microbial ecosystems, especially in host-associated microbiomes, is particularly appealing since in microbes the degree to which functional traits and phylogeny are linked (as opposed to unlinked by e.g. lateral gene transfer) remains to be determined^40^.

Future work should aim to establish whether or not the different populations within a phylogenetic core group present shared ecological functionality (e.g. via genome content analysis), and how such particular functionality relates to the overall ecosystem’s function and host well-being. It should also be established if these intra-core group populations exhibit competitive interactions, as could be expected by their plausible ecological redundancy and large intra-group diversity^41^. Yet another interesting line of research would be the assessment of the existence of social and rival clubs^42^ in the human gut pan-microbiome formed by different combinations of intra-core groups populations and(or) higher similarity clusters, as a road to better comprehend the rules that govern the ecological interactions that occur in the ecosystem. In this sense, the overall phylogenetic core would likely be partitioned into different structural possibilities should variables like age, gender, lifestyle etc. be taken into account.

## Conclusion

The present study shows that the human gut pan-microbiome contains a preeminent compositional phylogenetic core, defined in terms of discrete units of varying depth along the bacterial phylogeny, whose members are present in all individuals studied. Surely, the description of the human gut pan-microbiome phylogenetic core will be re-evaluated in the foreseeable future, eventually combining the use of improved phylogenetic grouping approaches, a more meaningful statistical sampling framework instead of using prevalence thresholds, larger and more comprehensive cohorts, deeper sequencing and longer sequence reads. Thus, the present description of the core should not be understood as an endpoint, but rather as a means to better understand the gut ecosystem, especially community assembly, as well as a guide for further research and experimentation. Our results led us to a new conceptual framework which we contend has great potential for advancing our understanding of microbial ecosystems, particularly host-associated microbiomes. In addition to providing a novel perspective on community assembly, the results derived from the present study should guide the selection of more meaningful combinations of bacterial species (or genomes) in many frequent *in vivo*, *in vitro*, or *in silico* experimental scenarios. Furthermore, the results presented in this work should be used as a revised list of “most wanted” bacteria to guide future genome sequencing^34,43–46^ and isolation^47^ efforts, especially as it also includes information on the biologically meaningful breadth of the targeted group’s pan-genome. Similarly, the development of microbiome-based therapies^48^ should also take into account the existence of the phylogenetic core.

## Electronic supplementary material

Supplementary Information

Datasets and Core groups’ statistics (.xlxs)
