# Supplementary material for: The human gut pan-microbiome presents a compositional core formed by discrete phylogenetic units
Source: Sci Rep. 2018 Sep 19;8:14069. doi: 10.1038/s41598-018-32221-8 (PMC6145917; doi:10.1038/s41598-018-32221-8)
Supplement: Supplementary file 1 — Supplementary Information [file 41598_2018_32221_MOESM1_ESM.pdf]

The human gut pan-microbiome presents a compositional core formed by discrete phylogenetic units

Daniel Aguirre de Cárcer[1*]

[1]Departamento de Biología, Universidad Autónoma de Madrid, Madrid, Spain.

Examples of conflicting results in the taxonomic assignment of core OTUs. For each selected core OTU, a Metadiversity Plot summarizes the taxonomic assignments obtained for its within-core OTU representative sequences. Label, color, and node sizes correlate with each specific taxon's preeminence in the OTU.

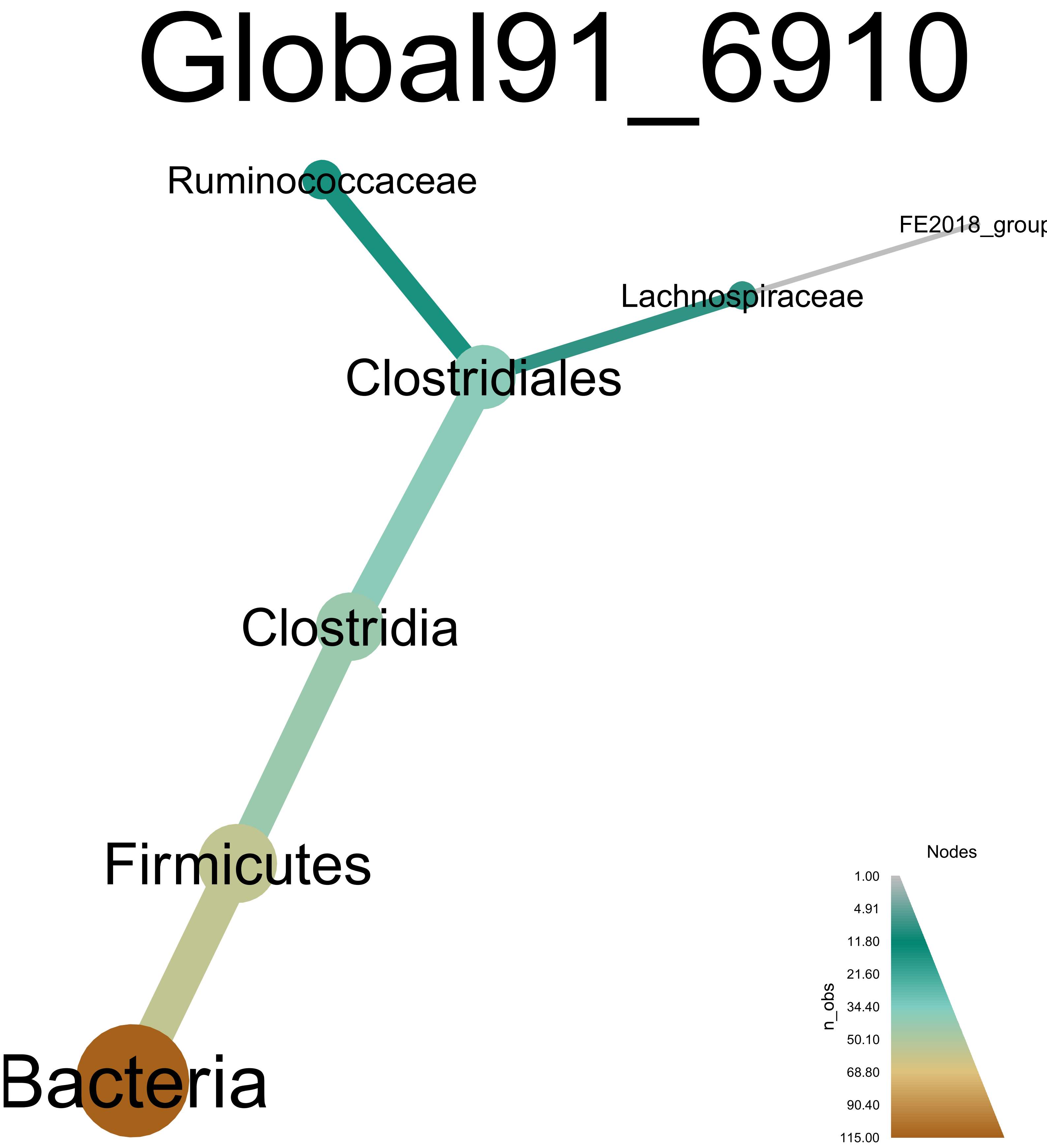

Global91_6910
Ruminococcaceae
FE2018_group
Lachnospiraceae
Clostridiales
Clostridia
Firmicutes
Bacteria
Nodes
n_obs
1.00
4.91
11.80
21.60
34.40
50.10
68.80
90.40
115.00

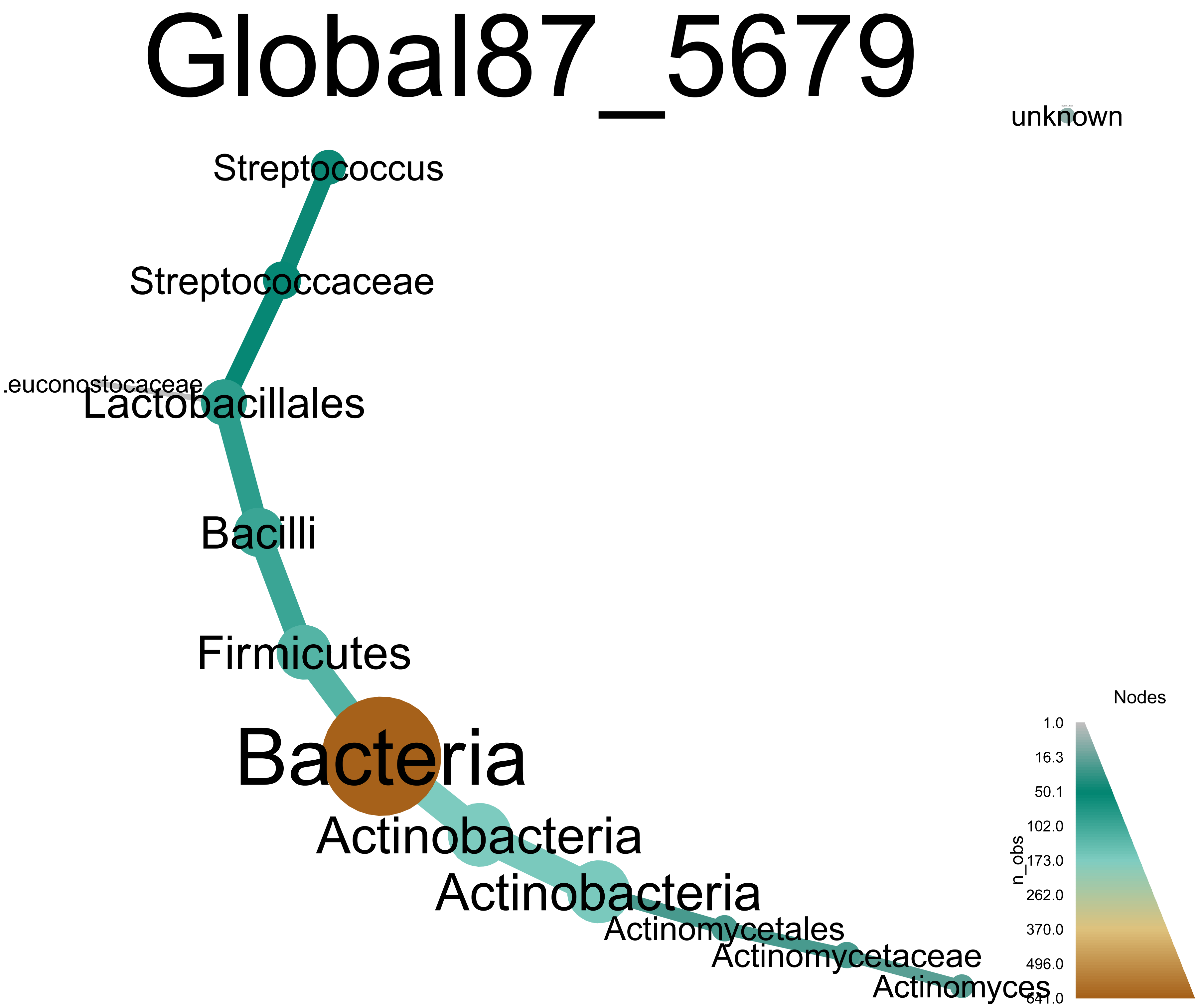
Global87_5679
unknown
Streptococcus
Streptococcaceae
.euconostocaceae
Lactobacillales
Bacilli
Firmicutes
Bacteria
Actinobacteria
Actinobacteria
Actinomycetales
Actinomycetaceae
Actinomyces
Nodes
n_obs
1.0
16.3
50.1
102.0
173.0
262.0
370.0
496.0
641.0
